# SENSING SPOUSAL CARE DYADS’ ACTIVITY PATTERNS: TWO NOVEL DYADIC METRICS FOR CAPTURING TIME USE

**DOI:** 10.1093/geroni/igae098.1413

**Published:** 2024-12-31

**Authors:** Lyndsey Miller, Wan-Tai Au-Yeung, Karen Lyons, Joel Steele, Jeffrey Kaye

**Affiliations:** Oregon Health & Science University, Portland, Oregon, United States; Oregon Health and Science University, Portland, Oregon, United States; Boston College, Chestnut Hill, Massachusetts, United States; University of North Dakota, Grand Forks, North Dakota, United States; Oregon Health & Science University, Portland, Oregon, United States

## Abstract

Spousal care dyads’ in-home activity and routines are typically captured through self-report. Remote passive sensing offers a sensitive, continuous, and ecologically-valid method of assessment that increases the ability to detect patterns of daily activities and function; however, there are challenges of deploying these technologies among dyads in the community setting and in the context of dementia. In this study of 47 co-habiting care dyads (person living with dementia and their spouse, x ®=71.3 years in age), we aimed to characterize aspects of daily dyadic activity patterns using an in-home sensor platform. Additionally, we aimed to understand the relationship between these novel activity patterns and the dyad’s relationship (caregiver burden and dyadic relationship strain). Activity measures included independent life space activity (dyads’ active time in separate rooms) and time spent out of the home, both of which were captured by infrared motion and door contact sensors. Dyads also wore an actigraphy watch that captured daily step counts (X ®= 3548/day) and nightly sleep duration (X ®= 7.6 hours). According to mixed effects modeling, there was a significant inverse association between the amount of time spent out of the home and the amount of time spent conducting independent life space activity (p < 0.001), but no significant associations with step counts or sleep duration. When relating activity patterns to aspects of the caregiving relationship, there was an inverse relationship between average step counts and level of caregiver burden (reported weekly), and between relationship strain and time spent out of the home.

